# 3D Approaches in Complex CHD: Where Are We? Funny Printing and Beautiful Images, or a Useful Tool?

**DOI:** 10.3390/jcdd9080269

**Published:** 2022-08-15

**Authors:** Adriani Spanaki, Saleha Kabir, Natasha Stephenson, Milou P. M. van Poppel, Valentina Benetti, John Simpson

**Affiliations:** 1 Department of Congenital Heart Disease, Evelina London Children’s Hospital, Guy’s and St Thomas NHS Foundation Trust, London SE1 7EH, UK; 2 School of Biomedical Engineering & Imaging Sciences, King’s College London, King’s Health Partners, St Thomas’ Hospital, London SE1 7EH, UK

**Keywords:** congenital heart disease (CHD), 3D imaging, echocardiography

## Abstract

Echocardiography, CT and MRI have a crucial role in the management of congenital heart disease (CHD) patients. All of these modalities can be presented in a 2D or a 3D rendered format. The aim of this paper is to review the key advantages and potential limitations, as well as the future challenges of a 3D approach in each imaging modality. The focus of this review is on anatomic rather than functional assessment. Conventional 2D echocardiography presents limitations when imaging complex lesions, whereas 3D imaging depicts the anatomy in all dimensions. CT and MRI can visualise extracardiac vasculature and guide complex biventricular repair. Three-dimensional printed models can be used in depicting complex intracardiac relationships and defining the surgical strategy in specific lesions. Extended reality imaging retained dynamic cardiac motion holds great potential for planning surgical and catheter procedures. Overall, the use of 3D imaging has resulted in a better understanding of anatomy, with a direct impact on the surgical and catheter approach, particularly in more complex cases.

## 1. Introduction

Congenital heart disease patients require precise and reliable imaging to facilitate decision making with respect to surgery, catheter intervention or ongoing review. There is currently a plethora of imaging choices available, including echocardiography, CT and MRI. All of these modalities can be presented in a 3D rendered format or interrogated by “slicing” images in user-defined imaging planes. Guidelines have been published for each of these modalities for the congenital heart disease patient [1,2,3,4]. The reader is referred to these publications for detailed review of each modality.

The use of a multiplanar approach permits detailed re-analysis of an imaging dataset in any desired plane, but does not present truly three-dimensional images. In contrast, 3D rendered images can be produced using ultrasound, CT and MRI data. However, the display of such images generally remains on a flat screen, with colour coding used to enhance depth perception. To overcome the limitations of this means of image display, 3D printing has gained traction to allow surgeons and interventionists to be able to have a static physical representation of the true cardiac anatomy. The aim of this paper is to review the key advantages of a 3D approach and to highlight potential benefits and limitations. We will be focusing on anatomical details rather than function assessment, and this will be performed with reference to specific lesions. Future developments to overcome remaining limitations will also be discussed.

## 2. Practical Approach

Echocardiography remains a dominant imaging modality in congenital cardiac imaging, due to its accessibility and high temporal and spatial resolution. CT and MRI both hold an integral part in congenital cardiology imaging, with different indications and strengths. One of the major disadvantages of echocardiography is that it relies on the patient’s acoustic windows. It is also restricted by a relatively limited field of view, which means that certain structures, especially extracardiac vasculature, are poorly visualised. CT remains relatively easily accessible compared to cardiac MRI, and can provide good quality images with high spatial resolution with shorter examination times using muti-detector scanners. The main concern with this modality is the use of ionising radiation, although this has been dramatically reduced with latest generation CT scanners. MRI imaging is limited by the need for longer breath holds and the fact that most sequences will need to be gated to the cardiac cycle. This can be challenging in paediatric patients, who might need sedation or general anaesthesia to achieve this successfully. Cardiac MRI, although time consuming and not readily available, remains an excellent radiation-free imaging modality providing robust data, especially for the sequential assessment of cardiac structure and function. The advantages and limitations of each imaging modality are summarised in Table 1.

## 3. Three-Dimensional Echocardiography

Echocardiography is frequently the only imaging modality used in congenital cardiac patients, even when surgical repair is required. Conventional 2D echocardiography necessitates mental reconstruction of the 3D image for each cardiac lesion. The way in which images are displayed in 2D is not intuitive to many surgeons and often the appearance does not resemble the anatomy as viewed intraoperatively. The limitations to 2D echocardiography become more evident in complex lesions, especially when compared to CT and MRI data.

By using 3D echocardiography, cardiac anatomy is depicted in all dimensions; while retaining adjacent anatomic landmarks to assist with orientation. The major advantage of this is that it provides clinicians with the depth of field that is missing in conventional echocardiography. Furthermore, in contrast to 3D CT and MRI, dynamic motion of the heart, including heart valves, is retained.

**Figure 1:**
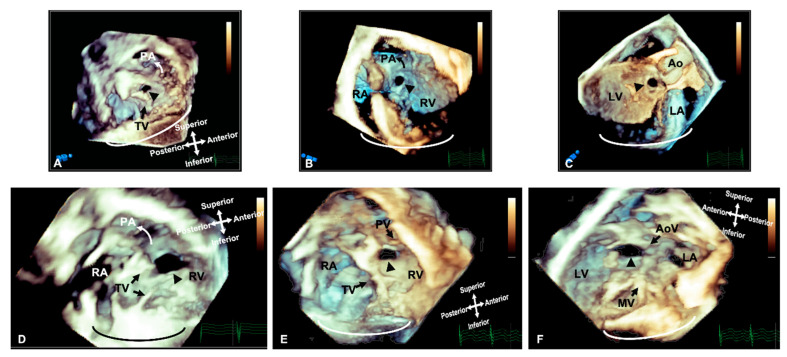
Three-dimensional rendered imaging demonstrating the appearance of VSDs of various morphologies. (**A**) Demonstrates a perimembranous VSD (arrowhead), as seen from the right ventricular view—here, we can see the area of proximity between the defect and the TV, which allows fibrous continuity between the AoV and the TV. An outlet muscular VSD (arrowhead) seen from the right ventricle and (**B**,**C**) left ventricle—the tissue surrounding the defect and the distance from the AoV makes it suitable for consideration of device closure. (**D**) A view from the right ventricle showing a large muscular VSD (arrowhead) positioned more anteriorly towards the pulmonary artery. A doubly committed VSD, as seen from the right ventricle (**E**) and left ventricle (**F**), demonstrating the proximity to the outlet valves, which allow fibrous continuity of the PV and AoV. All images are shown in anatomic orientation. Ao: Aorta; AoV: Aortic valve; LV: Left ventricle; MV: Mitral valve; PA: Pulmonary valve; PV: Pulmonary valve; RA: Right atrium; RV: Right ventricle and TV: Tricuspid valve.

This provides clinicians with better perspective of cardiac structures and allows for a more accurate evaluation of morphology, relative position, area, circumference and dynamic variation during the cardiac cycle [6] (Figure 1). This technique also offers non-conventional views, not available on 2D imaging [7], including the so called ‘en face’ view which mirrors the intraoperative surgical view and allows for rapid understanding of the pathology with surgeons’ and cardiologists’ collaboration in postprocessing [8].

**Figure 2:**
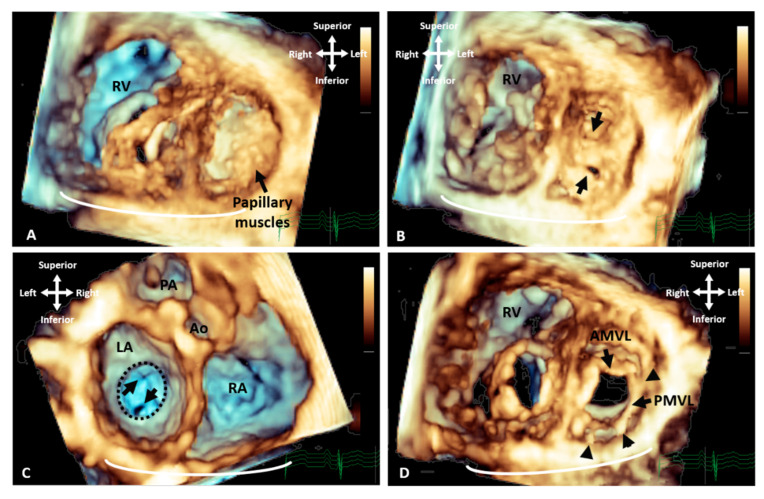
Three-dimensional TTE demonstrating the appearance of parachute mitral valve with complex subvalvar stenosis. (**A**) Demonstrates the thickened and fused subvalvar apparatus with indistinguishable papillary muscles and chords as seen from a distal ventricular view. A view through the middle of subvalvar tissue reveals two small orifices as seen from the (**B**) ventricular and (**C**) atrial aspects, the atrial view also shows the small annulus that is highlighted with the dashed line. (**D**) A view at the level of the mitral valve leaflets shows that although the posterior leaflet is tethered with multiple short chords (arrowheads), the anterior leaflet retains adequate excursion, with area of narrowing and stenosis being within the subvalvar tissue. Ao: Aorta; AMVL: Anterior mitral valve leaflet; LA: Left atrium; PA: Pulmonary valve; PMVL: Posterior mitral valve leaflet and RA: Right atrium.

There is extensive literature reporting the value of 3D echocardiography in specific congenital cardiac defects. It has proven most helpful for the evaluation of the mitral valve where it provides physiologic, morphologic and functional assessment [9]. Three-dimensional echocardiography is a more sensitive modality compared with 2D in identifying the lesion in the mitral valve apparatus responsible for pathology [10]. It demonstrates the interaction of the valve leaflets, and the geometry of the sub-valvar support apparatus with superior delineation of chords and papillary muscles [11] (Figure 2). The addition of colour to 3D imaging can further facilitate identification of the regurgitant orifice and provides additional quantitative information.

**Figure 3:**
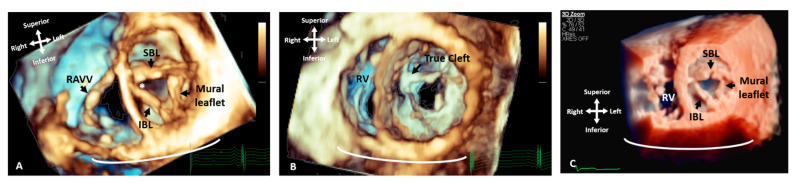
Three-dimensional TTE comparing the appearance of a LAVV and a true mitral valve cleft. (**A**) A LAVV demonstrating the zone of apposition (asterisk) compared to (**B**) a true cleft of the AMVL, both demonstrated with rendered 3D imaging and (**C**) illustrates the greater degree of resolution that can be appreciated in the structures with True Vue imaging modality. The images are depicted in anatomical orientation from the ventricular aspect. AMVL: Anterior mitral valve leaflet; IBL: Inferior bridging leaflet; RV: Right ventricle; RAVV: Right atrioventricular valve and SBL: Superior bridging leaflet.

Similar advances have been noted in the management of AVSD spectrum defects, features of which are sometimes difficult to appreciate in 2D imaging (Figure 3, Video S1). Three-dimensional echocardiography can demonstrate abnormal insertion of the anterolateral papillary muscle, abnormal chordal insertion to the superior bridging leaflet, imbalance of superior and inferior bridging leaflet size, and commissural abnormalities [7]. This information can facilitate surgical planning and identify whether the closure of the zone of apposition in the left AV valve is indicated to prevent AV valve failure [12].

**Figure 4:**
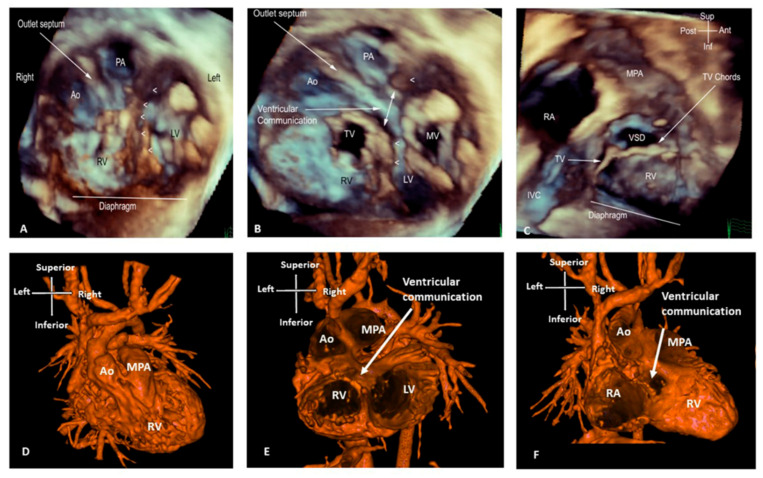
Three-dimensional TTE and CT demonstrating the intracardiac anatomy in a case of DORV. (**A**)View from ventricular apex, demonstrating the position of the ventricular septum (small arrowheads) in relation with the great arteries, both committed to RV. (**B**) Similar view to A, cropped closer to the base of the heart, depicting the interventricular communication. Baffling the aorta to the LV can be established without impinging on the tricuspid valve or obstructing the pulmonary artery. (**C**) Right ventricular view of interventricular communication, looking at the IVS “en face”. The rims of the defect can be appreciated in relationship to the pulmonary outflow, TV and chords. (**D**) View of the anterior surface of the heart, demonstrating that both great arteries are committed to RV. (**E**) Similar view to B (from ventricular apex), demonstrating the relationship of the great arteries and the interventricular communication. (**F**) Similar view to C (“en face” view of IVS from RV side), illustrating how the aorta can be baffled to LV. Ao: Aorta; IVC: Inferior vena cava; LV: Left ventricle; MPA: Main pulmonary artery; MV: Mitral valve; PA: Pulmonary artery; RA: Right atrium; RV: Right ventricle; TV: Tricuspid valve; VSD: Ventricular septal defect.

There is also a clear role for 3D echocardiography in delineating anatomy and surgical planning in patients with double outlet right ventricle; when the ventricles are balanced and a biventricular repair remains the desired approach (Figure 4, Video S2). Three-dimensional rendered images can demonstrate the size of interventricular communications and its relationship to the origin of the great arteries and also identify factors precluding biventricular repair, such as straddling chords. This approach assists in assessment of the anatomy to determine whether baffling the left ventricular output to the aorta would be feasible, whether an RV to PA conduit is required, or whether the anatomy is more suitable for an arterial switch operation [13].

**Figure 5:**
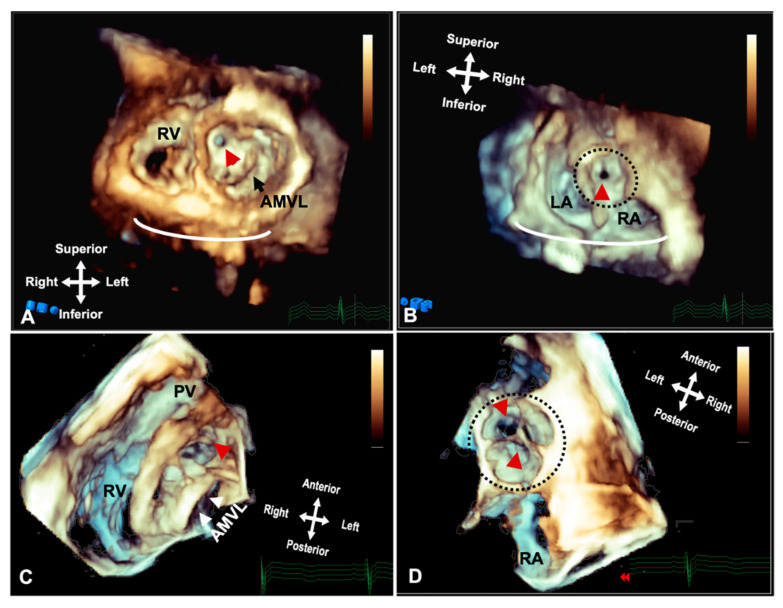
Three-dimensional rendered imaging from TTE/TOE demonstrating the appearance of significant subaortic stenosis. (**A**) TTE demonstrates a thickened circumferential ridge within the LVOT causing significant stenosis and leaving a small orifice (arrowhead) shown from the ventricular aspects and (**B**) the atria, with the aorta highlighted by a dashed line. (**C**) Shows a subaortic membrane as seen from the ventricular aspect and (**D**) from the atria with the circumferential membrane seen through the leaflets of the aortic valve highlighted by the dashed line. AMVL: Anterior mitral valve leaflet; LA: Left atrium; RA: Right atrium and RV: Right ventricle.

The use of 3D echocardiography in patients with complex left ventricular outflow tract obstruction can provide additional details on the obstructive substrate (Figure 5, Video S3). Volume rendered data from different perspectives including projection through the aortic valve combined with view from the ventricle and a long axis view of the left ventricle, provide an accurate demonstration of the obstructive lesion and relevant surrounding structures. Identification of the mechanism of obstruction is crucial before surgery, as surgical resection is frequently performed through the aortic valve which affords a restricted view and subsequently extensive resection may lead to significant aortic valve regurgitation postoperatively [7].

**Figure 6:**
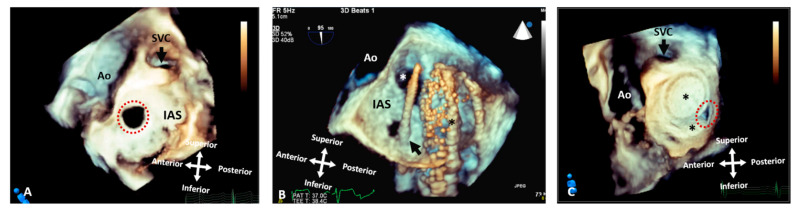
Three-dimensional rendered images from TOE demonstrating the appearance of defects within the atrial septum. All images are shown in anatomic orientation from the left atrial view. (**A**) Demonstrates a secundum ASD as outlined by the dashed line, which appears ideal for closure with a percutaneous device. (**B**) This image illustrates careful intra-procedure imaging depicting a septum with multiple defects. Live 3D can be very informative in allowing visualization of catheters (arrow), multiple defects (white asterisk) and balloons used to interrogate the defect size and septal tissue (black asterisk). (**C**) Two ASD occlusion devices (asterisk) used to close multiple defects, with a small residual defect noted posteriorly (dashed line). Ao: Aorta; IAS: Interatrial septum; LA: Left atrium; RA: Right atrium; RLPV: Right lower pulmonary vein; RUPV: Right upper pulmonary vein and SVC: Superior vena cava.

3D TOE provides additional information during elective or pre-procedural imaging of selected patients and has led to advances in interventional procedures. There is an ever-growing group of TOE-guided procedures where 3D echocardiography facilitates both appropriate patient selection and also intra-procedural guidance and decision making. Real-time 3D imaging is recommended for visualization of catheters, delivery systems, and identifying device position, any residual shunt and the relationship of the device to adjacent structures [4]. Three-dimensional TOE has an established role in ASD device closure, since it can provide a precise delineation of the anatomic defect and investigate its suitability for device closure [14] (Figure 6). One study has demonstrated 54% reduction in total fluoroscopic time and 78% radiation reduction in ASD closures using 3D TOE guidance with no associated complications [15].

**Figure 7:**
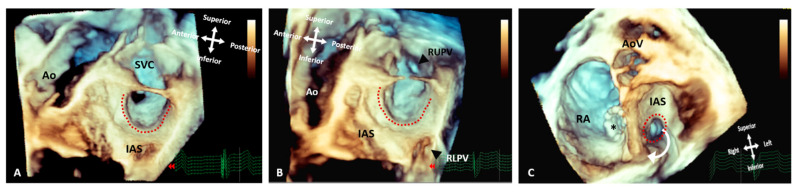
Three-dimensional rendered images from TOE demonstrating the appearance of Superior Sinus Venosus ASDs before and after stenting. (**A**) Demonstrates a superior sinus venosus ASD as outlined by the dashed line–seen from the left atrial aspect. (**B**) Illustrates that careful imaging and angulation allows visualization of the right pulmonary veins (arrowheads), the anomalous RUPV and the RLPV draining into the LA. (**C**) An image seen from the ventricle with the AV valves removed allowing visualization of the roof of the atria. A covered stent in situ within the SVC extending into the RA (asterisk) and the unoccluded defect (dashed line) allowing the anomalous vein to drain around the stent back into the LA. All images are shown in anatomic orientation. Ao: Aorta; AoV: Aortic valve; AS; Interatrial septum; LA: Left atrium; RA: Right atrium; RLPV: Right lower pulmonary vein; RUPV: Right upper pulmonary vein and SVC: Superior vena cava.

Similarly, 3D TOEs are used to guide percutaneous VSD closure as well as paravalvar and baffle leak closure, where an ‘en face’ view of the defect is feasible, making the location of the leak more easily identifiable [16] Another interventional procedure that has been made feasible through 3D imaging and especially 3D TOE guidance is the transcatheter closure of sinus venosus ASDs which are usually associated with partially anomalous pulmonary venous return of the right sided upper and or middle pulmonary veins (Figure 7, Video S4). In a single centre study, >75% of patients referred for transcatheter correction by placing a covered stent into the SVC were successfully treated, rendering this technique an effective and reproducible alternative to surgical closure [17]. Transoesophageal 3D echocardiography is used to assess the defect and the relationship of the right pulmonary veins to the superior vena cava–right atrium junction during stent deployment, and to assess the position of the deployed stent as well as potential residual shunts [18].

## 4. CT

CT provides extremely high-resolution cardiovascular imaging, including 3D rendered views, with the most recent generation of ultrafast scanners reducing both scan time and radiation dose. This facilitates high-resolution imaging in the context of un-cooperative or unstable patients, for example in an intensive care setting. However, CT still uses ionising radiation and paediatric patients are likely to need repeat imaging and their rapidly dividing cells make them more susceptible to radiation damage [19]. It is also known that CT alongside angiography are the main sources of radiation exposure during follow up of adult patients with congenital heart disease [20]. Due to the lifelong sequalae involved, CT scanners have incorporated radiation dose reduction technology and dose optimisation protocols to minimise associated risks [5]. CT acquisition parameters are adjusted to minimise exposure, and post-processing filters are applied to reduce noise and maintain image quality [21,22]. Dual-energy CT (DECT) is a new technology that is able to reduce the radiation exposure dose even further [23].

**Figure 8:**
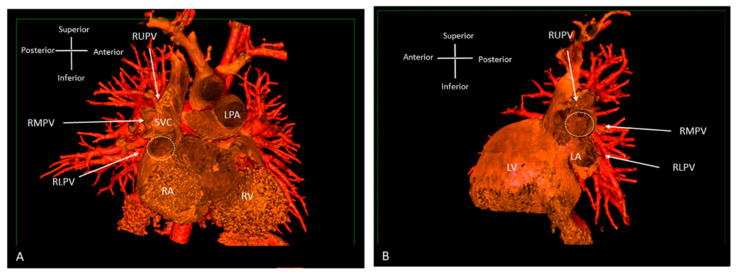
Three-dimensional rendered images from CT, demonstrating the appearance of Superior Sinus Venosus ASD. (**A**) Demonstrates a superior sinus venosus ASD as outlined by the dashed line—seen from the right atrial aspect. RUPV and RMPV drain anomalously to the SVC. RLPV can be seen draining to LA. (**B**) Depicts a superior sinus venosus ASD from the left atrial aspect, allows imaging of the anomalous drainage of the RUPV and RMPV to the SVC. RLPV illustrated draining into the LA. LA: Left atrium; LPA: Left pulmonary artery; LV: Left ventricle; RA: Right atrium; RV: Right ventricle; SVC: Superior vena cava; RLPV: Right lower pulmonary vein; RMPV: Right middle pulmonary vein; RUPV: Right upper pulmonary vein.

CT offers the option of both surface and volume rendering, with surface rendering using only part of the available data for 3D reconstruction (Figure 8). After the determination of surface representation, this can be a fast technique with good depth perception, but it relies on well-differentiated surfaces and thresholds [5]. The introduction of dynamic CT has allowed for the uninterrupted acquisition of cardiac structures, resulting in even shorter scanning times.

The main advantage of CT is the imaging of complex vascular structures. In patients with Tetralogy of Fallot, 3D CT data demonstrates pulmonary arterial distribution, with the anatomy of the right ventricular outflow tract and any potential coronary artery anatomy variations precluding or complicating surgical or interventional procedures. Three-dimensional CT imaging provides a comprehensive delineation of the pulmonary blood supply in patients with pulmonary atresia and major aortopulmonary collaterals, allowing for the planning of unifocalisation and pulmonary artery reconstruction. Three-dimensional aortic arch reconstructions can accurately demonstrate the arch anatomy and length of the narrowed segment in complex cases of coarctation, as well as the relationship with head and neck vessels [5]. The increasing detection of vascular rings due to comprehensive foetal screening has resulted in the development of specific 3D CT protocols for these vascular structures, combined with an airway assessment via endoscopic bronchoscopy to allow for early recognition of any potential for compression [24]. In patients being considered for complex biventricular repair, such as unbalanced AVSD or double outlet right ventricle, 3D CT can depict complex intracardiac anatomy by identifying great artery relationships, ventricular septal defect size/location and some information on the atrioventricular valves (Figure 4). Some types of repairs involve baffling the blood flow from the left ventricle to the aorta, and CT can assist in the assessment of feasibility and an estimation of the post-repair size of the remaining ventricular cavity.

## 5. MRI

3D MRI acquired data can produce whole heart imaging and visualise the extracardiac vasculature and relationships to adjacent structures while remaining free of radiation. Contrast-enhanced angiography and non-contrast enhanced 3D whole heart imaging can be used as required to interrogate cardiac anatomy on a multiplanar reformat platform [5]. A new offline 3D planning method has been compared to conventional 2D MRI imaging [25]. The anatomical coverage was of equal imaging quality, using both methods. However, the 3D offline tool was more time efficient, which is particularly relevant in patients under general anaesthesia or sedation.

**Figure 9:**
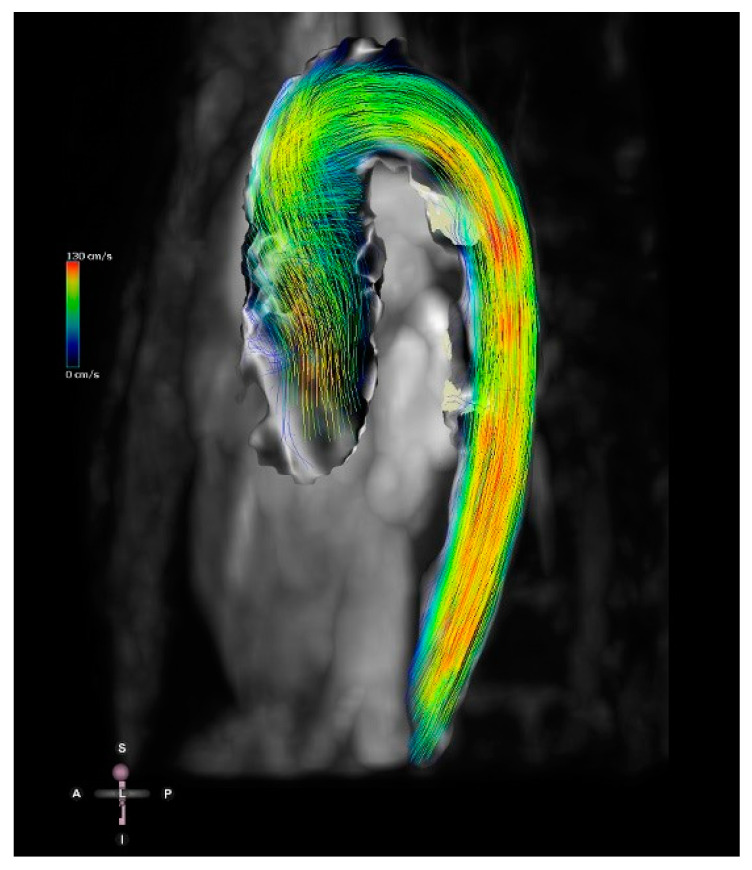
Four-dimensional flow image of a patient with a bicuspid aortic valve and dilation of the ascending aorta. Streamlines show the 4D flow dataset in mid-systole. Streamlines represent the direction of the flow and are colour-coded for velocity.

The recent development of 4D flow MRI includes a phase contrast MRI study with flow encoding in all three spatial directions relative to all three dimensions of space and to the dimension of time throughout the cardiac cycle [26] (Figure 9). This allows for volume flow quantification with the added advantage of enabling the retrospective calculation of blood flow through any plane of interest within the 3D volume acquired.

Simulations using computational fluid dynamics (CFD) derived from MRI data and patient-specific 3D models offer the possibility for modelling circulation in congenital heart defects, and can provide measures of flow and how these would be affected by interventions that change the geometry [27]. A clinical application of current methods remains limited, mainly due to long computation times and complexity. A newer lean steady state simulation technique has been recently described, with fast and reliable results in patients with physiologically univentricular hearts [27].

## 6. Three-Dimensional Printing

**Figure 10:**
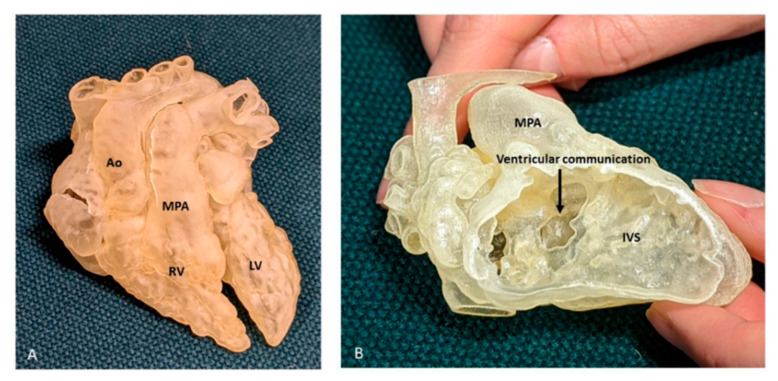
Three-dimensional printed model from a case of DORV. (**A**) View of the anterior surface of the heart, demonstrating the relation of the great arteries, both committed to RV. (**B**) Right ventricular view of interventricular communication, looking at the IVS “en face”. The rims of the defect can be appreciated, but there is paucity of any additional intracardiac details, especially with regard to TV. Ao: Aorta; LV: Left ventricle; MPA: Main pulmonary artery; RV: Right ventricle.

Three-dimensional printing is used in patients with congenital heart disease to precisely visualise complex anatomy, and to depict complex intracardiac spatial relationships (Figure 10). The majority of cardiac 3D printing is performed from contrast-enhanced CT and MRI datasets, which in general produces high-resolution images of most intracardiac structures [28]. There is currently an increasing use of 3D models from echocardiographic data, with very good accuracy, although there are technical limitations due to lower spatial resolution, stitch artifact and lower signal to noise ratio, when compared to MRI and CT [28]. Three-dimensional printed models are starting to have an established role in the management of patients with CHD, and the Radiological Society of North America 3D Printing Special Interest Group recently established guidelines framing the clinical indications for the use of these models and the best approach for imaging acquisition, segmentation tools, 3D printing and post-processing the models [29]. Although gaining traction, the production of 3D printed models involves segmentation of the imaging data prior to printing, which is time-consuming and introduces a source of “human” error. In addition, the models produced are static and rarely include all valvar structures, and interrogation of the model can involve cutting of the printed model.

3D printing is useful in defining the most appropriate surgical management in specific cardiac lesions. A landmark multi-centre European study has demonstrated a change in the management or surgical approach after a 3D model was reviewed in 19 out of 40 complex cases, when compared to multidisciplinary consensus prior to access to the model [30]. In 10 of these cases, an inspection of the 3D model resulted in significant modifications of the surgical plan; for example, changing from univentricular to biventricular repair. Some of these lesions were previously regarded as being unrepairable anatomy, and they underwent successful univentricular or even biventricular repair. In other cases, the surgical approach was modified to reduce the potential for surgical complications. Three-dimensional printing can also be a valuable tool to plan extracardiac surgery in patients with congenital heart disease, such as high-risk unifocalisation surgery in patients with pulmonary atresia and major aortopulmonary collateral arteries. Three-dimensional models can also provide explicit information regarding the patients’ airways. It is widely known that in congenital patients, the airways may need to be included in surgical planning, as they may be directly affected by aberrant or dilated vessels. Three-dimensional printing recently led to a significant revolution in the management of these patients, with the use of 3D printed bioresorbable airway splints [31].

There is currently no imaging modality that can demonstrate all the anatomical features necessary in complex lesions to guide surgical planning. A 3D hybrid printed model combing MRI/CT and 3D echocardiographic data can provide diagnostic information superior to individual imaging modalities and aid in hands-on simulations of the optimal surgical approach in challenging cases such a straddling AV valves [32]. Combining the strengths of different imaging techniques is now feasible and permits the ability to print hybrid 3D models of cardiac lesions [33].

## 7. Foetal Cardiac Techniques

Antenatal detection of congenital heart disease has become increasingly common with the development of appropriate foetal screening, including more extensive visualisation of the great arteries in the upper mediastinum. This is based on 2D foetal echocardiography, which can sometimes be challenging, due to certain foetal and maternal factors. Three- and four-dimensional foetal echocardiographic techniques, such as spatiotemporal image correlation imaging (STIC), can be extremely helpful for more complex lesions such as double outlet right ventricle [34] or vascular rings [35]. However, all echocardiographic techniques are constrained by acoustic windows, which will be limited by foetal lie, acoustic shadow and maternal factors. The reliability, consistency and practical benefits of these techniques remain uncertain [36].

**Figure 11:**
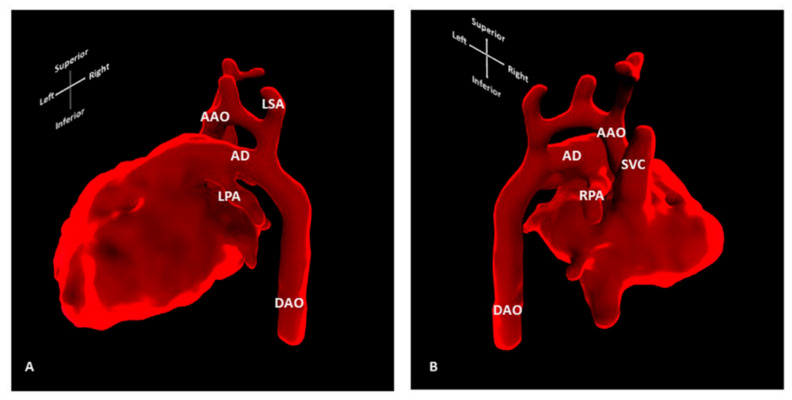
Three-dimensional foetal cardiac MRI showing high suspicion of CoA. Segmentation of motion-corrected 3D foetal cardiac MRI data, showing (**A**) left lateral and (**B**) right lateral view of the foetal heart. Foetal cardiac MRI was conducted at 30 + 2 weeks’ gestation for suspected coarctation of the aorta. Neonatal coarctation requiring surgical repair was confirmed after birth. AAO: Ascending aorta; AD: Arterial duct; DAO: Descending aorta; LPA: Left pulmonary artery; LSA: Left subclavian artery; RPA: Right pulmonary artery; SVC: Superior vena cava.

**Figure 12:**
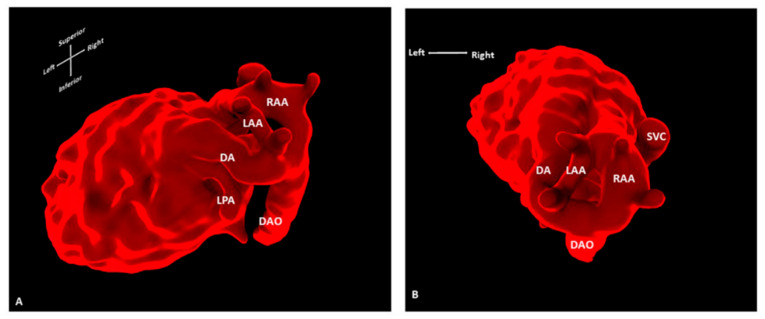
Three-dimensional foetal cardiac MRI showing a double aortic arch. Segmentation of motion-corrected 3D foetal cardiac MRI data, showing (**A**) superior and (**B**) oblique left lateral/superior view of the foetal heart. Foetal cardiac MRI was conducted at 32 + 4 weeks’ gestation for suspected double aortic arch. Surgical findings confirmed the presence of a smaller left aortic arch in addition to dominant right aortic arch and left arterial duct. AD: Arterial duct; DAO: Descending aorta; LAA: Left aortic arch; LPA: Left pulmonary artery; RAA: Right aortic arch; SVC: Superior vena cava.

Standard 2D foetal (cardiac) MRI is susceptible to foetal and maternal motion [37]. Novel techniques using motion-corrected slice-volume registration software now allow for successful 3D whole heart imaging, including an assessment of extra-cardiac vasculature and blood [38,39]. Motion-corrected black-blood 3D volumes exhibited good spatial agreement when compared with paired ultrasound data, and in 10 of 85 foetuses reported, new anatomical features were described that had previously been undetected, all of which were confirmed postnatally [37]. Three-dimensional foetal cardiac MRI is currently applied in many settings, including the refinement of the prenatal diagnosis of coarctation of the aorta [40] (Figure 11), and the provision of additional information on vascular rings [41] (Figure 12). In addition, the characterisation of extracardiac pathology such as pulmonary lymphangiectasia on foetal MRI can provide useful information for assisting counselling and postnatal management, as it is an adverse prognostic factor in cases with hypoplastic left heart syndrome [42]. In our institution, foetal cardiac MRI has been approved for applications in clinical practice, and offers a safe and highly complementary adjunct to ultrasound in the antenatal diagnosis of congenital heart disease.

## 8. Fusion of 3D Imaging Modalities

We have already highlighted the advantages of 3D TOE in guiding percutaneous procedures. Three-dimensional echocardiography has also allowed for fusion imaging, where the echocardiographic and fluoroscopic images are synchronised and displayed in the same visual perspective to provide a better spatial relationship of the intracardiac anatomy [7]. The TOE probe position and orientation are co-registered with the fluoroscopy image, with markers being applied to important structures on the 3D images, which are then concurrently shown in fluoroscopy, thus assisting with in interventions [33]. This type of fusion imaging addresses some of the pitfalls associated with fluoroscopy, such as a poor visualisation of soft tissue and the use of 2D displays only, while remaining a non-contrast and non-radiation technique. At present, the most common applications of this technology include the deployment of TAVR, especially in patients with non-calcified valves and paravalvular leak, atrial septal defect and left atrium appendage closure [43]. Preliminary data in echocardiographic–fluoroscopic imaging in children with congenital heart disease suggest that this technique is feasible and safe in guiding interventional procedures, such as atrial septal defect and ventricular septal defect closure, and aortic valve dilatation and right ventricular outflow tract revalvulation [44].

The feasibility and spatial accuracy of pre-procedural 3D CT and MRI images to 3D rotational fluoroscopy registration to guide interventional procedures in patients with congenital heart disease has also been investigated. This technology displays great potential in integrating three-dimensional imaging in the catheterisation laboratory to reduce procedure time, radiation and contrast dose [45]. Assessing coronary arteries remains relevant in patients with congenital heart disease, and recent work fusing resting 3D echocardiography-derived myocardial deformation with coronary CT angiography permits a simultaneous assessment of the degree of coronary stenosis and its haemodynamic significance. This combined evaluation of coronary anatomy allows for an evaluation of the presence and extent of myocardial ischaemia without the need for the mental reconstruction of coronary arteries and their territory [46].

## 9. Extended Reality

**Figure 13:**
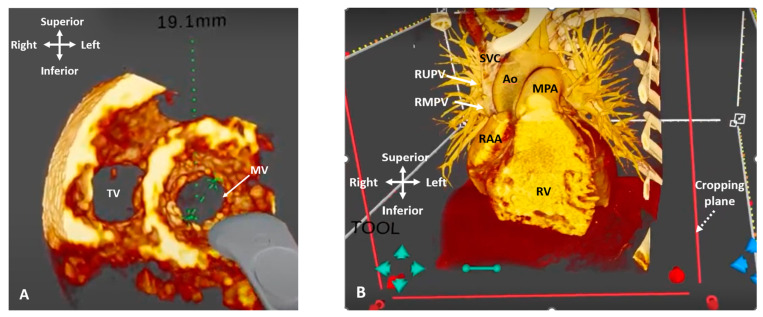
Three-dimensional rendered imaging in virtual reality. (**A**) 3D TOE image viewed from ventricular apex, demonstrating view of the mitral and tricuspid valves. A measurement of the mitral valve annulus has been performed using the in-built tool. (**B**) CT demonstrating a case of Superior Sinus Venosus ASD. The cropping plane (dashed arrow) is cropping into the volume from the anterior plane and demonstrating the entry points of the RUPV and RMPV into the SVC (arrows). Ao: Aorta; MPA: Main pulmonary artery; MV: Mitral valve; RAA: Right atrial appendage; RMPV: Right middle pulmonary vein; RUPV: Right upper pulmonary vein; RV: Right ventricle; SVC: Superior vena cava; TV: Tricuspid valve.

Extended reality (XR) denotes any technology combining a computer-generated environment with the real world, and is usually represented as a spectrum, from complete immersion in a digital world to physical reality [47]. It encompasses virtual, augmented and mixed reality, as well as other holographic displays. These techniques are usually achieved using a specialist head-mounted display (HMD) and body-tracking technology which enable realistic 3D depth perception and human–computer interaction, where the virtual world responds to user cues via controllers or gestures. Following on from advances in graphics technology and computer processing power, XR environments can offer 3D representations of cardiac anatomy and provide an interactive platform, whereby the surgeon or interventionalist can manipulate and rotate images to their own perspective in all directions (Figure 13). Additionally, XR can overcome the disadvantages of other 3D technologies by being able to represent dynamic structures such as valves and their support apparatuses, and by avoiding the additional time and cost of creating physical 3D printed models. An interrogation of the images using cropping planes and the measurement of cardiac structures is also possible in the XR environment.

XR holds great potential for the planning of surgical and catheter procedures in congenital heart disease, where an understanding the spatial relationships of structures is key. Ye et al. demonstrated a reduced planning time (51.65 ± 11.11 min vs. 65.71 ± 18.07 min; *p* < 0.05) and an improved delineation of anatomy in 34 patients with DORV, where a pre-operative XR review of CT imaging was performed [48]. In a single-centre retrospective study, the potential impact of the presentation of 3D echocardiographic data, in addition to conventional 2D/3D echocardiograms, in patients who had undergone atrioventricular valve repair, was evaluated. In 67% of cases presented with VR, surgeons reported having an added confidence in their understanding of each patient’s pathology. They also highlighted that they would have made minor modifications to the surgical approach in 53% and major modifications in 7% of cases [49]. Similar to 3D printed models, XR has shown feasibility in assisting with device sizing and implantation in cases such as the transcatheter correction of sinus venosus ASD [50] and the plugging of paravalvar leaks [51], although further work is required to show benefits over the current planning methods.

New advances in lightweight, transparent HMDs and hand gesture control have made 3D intra-procedural guidance possible. Augmented reality (AR) was used to display CT imaging during 17 complex pulmonary artery surgical repairs in children [52]. This study showed that this was a feasible technique in the operating room, and there were no complications related to use of the system, but it did not describe any outcome measures related to its usefulness compared to standard imaging. Another group demonstrated that an AR guidance system that displayed CT data intra-procedurally enabled the placement of cerebral embolic protection devices in six patients undergoing TAVR without the need to perform aortic arch angiograms [53]. This study showed how XR image-guidance could reduce the radiation and contrast dose in vulnerable groups. See-through HMD technology is still in its infancy, and the headsets at present are very expensive and have limitations such as a narrow field-of-view, which may limit uptake in clinical practice. Work continues on further improving the graphics and tracking technology within HMDs, as well as through other XR media such as holographic screens and laser interferography-based ‘holograms’ [54], although further work is required to show feasibility and benefit within the clinical workflow.

## 10. Reverse Engineering

3D models can facilitate interventional planning by allowing interventionalists to simulate and size percutaneous valves or occluder devices, when the commercially available sizes have not been ideal. This applies to septal defects, but is particularly useful in patients with Tetralogy of Fallot, where the dilation of the right sided structures can pose technical challenges to valve implantation [55], as well as in patients with coronary fistulae with convoluted course and unpredictable drainage [56].

Patient-specific 3D printed models have been used in the development of new techniques, such as the transcatheter correction of sinus venosus ASD and partially anomalous pulmonary venous return via the implantation of a covered stent [17]. Suitability for closure was assessed using a multimodality stepwise approach, including 2D and 3D cross-sectional imaging and ex vivo simulation with printed or virtual 3D models. In carefully selected patients, custom made stents were produced. During simulation, potential residual shunt or obstruction was also assessed. Transcatheter correction with simultaneous TOE was performed in 25 patients with no mortality, suggesting that this technique can be considered as an alternative to surgery [17].

A new technique providing personalised external aortic root support in patients with Marfan’s syndrome has also been made feasible, using spatial MRI and CT data to create a computer aided design and a physical model for each individual patient [57]. A replica of the aorta is used as a former on which a macroporous external support is manufactured prior to surgery. The first operation was performed in 2004, and it has now undergone Health Technology Appraisal by the British National Institute for Health and Care Excellence [58].

## 11. Teaching/Training/Patient Information-Engagement

3D cardiac imaging, and especially printed models, appear to be gaining an integral role in surgical and interventional training. These models provide a great tool for simulation, allowing surgeons to practice surgical techniques and expose them to a variety of congenital defects in a structured away, instead of leaving all of the technical training to the operating theatre. There is data suggesting a significant reduction in time required for specific procedures after performing simulation surgeries in 3D models [59].

The role of these models extends to the teaching of healthcare professionals, including nurses and doctors in training. Having access to these physical or digital replicas of the cardiac anatomy has been shown to be a great educational tool, offering experiential learning to medical personnel. Recently, an open-source library to share stereolithography files, ready to print across all of the Association for European Paediatric and Congenital Cardiology members has been created, providing access to a wide spectrum of cardiac pathologies [55].

Three-dimensional models have also been used when counselling patients, with a proven enhancement in patient’s understanding and subsequent compliance without impacting on clinical workflow [60]. Patients, including parents of paediatric patients, report that their perceived understanding with digital models was significantly higher than with the conventional ones. Their use has also been investigated in the setting of transition clinics, with very encouraging comments from teenagers who report finding the models both interesting and helpful in understanding their disease, and alleviating their anxiety [61].

## 12. Challenges

There are clear benefits that have already been highlighted with regard to the use of 3D cardiovascular imaging. It provides the depth of field needed to fully appreciate the cardiac anatomy, and it allows users to interrogate live or saved images while providing the opportunity to re-interrogate stored data, as clinically indicated. It is a valuable educational tool, providing a new insight into congenital lesions and facilitating a more user-friendly method of imaging the heart without the need for a mental 3D reconstruction of the conventional 2D image.

One of the main challenges of 3D echocardiography is the current lack of consistency in image orientation, although there are guidelines in place. Using the same alignment as is used with other imaging modalities, and to display images in an anatomically correct orientation is recommended [62]. The coherent and reproducible use of 3D echocardiography to display images not previously used in traditional 2D echocardiography requires application, and there is a learning curve. This highlights the need for a teaching program by echocardiographers who are well versed in 3D imaging, with multiple formative assessments and continuous guidance, both in volume acquisition and volume rendering. Gaining the skills required is a time-consuming process, but once mastered, the time needed to acquire and display the information will be reduced to an acceptable workload.

There are also additional considerations in children, such as higher heart/respiratory rates and overall smaller cardiac structures. Three-dimensional echocardiography might be technically more challenging, due to a lack of co-operation, with some patients requiring sedation to achieve optimal imaging with no stitch artifact. While higher frequency probes are recommended for children, 3D imaging is usually acquired with wider bandwidth adult probes, whose footprints and lower frequencies might be suboptimal for these patients [8]. The above factors make the adoption of a standardised protocol in this patient population difficult. Volume acquisition necessitates excellent image optimisation skills, as well as the flexibility to capture a volume at any point during the study, with appropriate and rapid changes between the 2D and 3D probes. In procedure guidance, which uses TOE imaging, an important limitation in the paediatric population remains the lack of availability of a smaller sized 3D probe that is suitable for patients of this size.

One of the limitations of 3D printing is that it remains an expensive imaging technique, currently used in specialised congenital cardiac centres, and requiring in depth training and specific expertise and resources. With appropriate protocol modifications, it seems possible to print high-quality and affordable 3D printed cardiac models that have been useful for improving the quality of care of the patients [63]. There also needs to be a thorough system in place allowing for the appropriate storage and maintenance of the physical models. There are currently 3D printed models using echocardiography data, but there are fundamental differences in the modality, image display, segmentation and printing technology that make 3D printing from echocardiography more challenging than printing from CT or MRI [64]. Echocardiography presents significant advantages since it has lower cost, no radiation, high temporal resolution and remains easily accessible.

3D printed models are not dynamic and cannot be re-cropped once produced. With respect to their use in catheter interventions, they do not respond to balloons and stents in the same way as native tissue, and they cannot accurately represent the physiological settings of these procedures [65]. It might be that the printed model is not representative of the patient’s anatomy as the pathophysiology evolves, and specifically in children as patients grow; both of these factors result in significant structural changes. This underlines the need for suitably timed imaging and 3D model printing prior to any planned procedure.

## 13. Future

Although the introduction of 3D cardiovascular imaging has enhanced our understanding of congenital heart disease, there remain areas where further comprehension is required to optimise patient management. Three-dimensional imaging has offered a new insight into our understanding of the mechanism of tricuspid valve regurgitation in hypoplastic left heart syndrome [66,67]. Similar findings have been reported in patients with complete AVSD, where the dynamic shape and motion of the common AV junction has been visualised and quantified [66]. A more profound understanding of the mechanisms of valve failure in these patients may facilitate novel surgical strategies or medical therapies [7].

One of the current limitations of 3D TOE in the paediatric population is the size of the adult 3D probe, which can be used ideally in patients >25kg. The development of paediatric miniaturised 3D TOE probes will enable the use of this modality in more patients, and could result in more TOE-guided procedures. This pitfall might also be addressed via the use of intracardiac echocardiography, a modality that is increasingly being adopted to guide percutaneous cardiac structural interventions. This was previously limited by the presence of only two-dimensional imaging planes, but a three-dimensional system has been assessed with encouraging results in procedure guidance [68]. Additional work is required so that the technical characteristics of this modality permit wider and more meaningful use in the catheter laboratory.

There is a constant evolution of 3D printing technologies, as well as the materials used. With the help of tissue engineering, biocompatible and human-like tissue materials are currently being developed. Surgical patches and parts of the heart are close to being manufactured, while aiming to be able to fabricate complete and functional hearts [63].

There is no doubt that the use of 3D imaging has resulted in high quality imaging in congenital heart disease, as well as a better understanding of structural abnormalities. What still remains to be answered is whether the introduction of this additional dimension is consistently leading to better patient outcomes. A patient-specific surgical approach could result in shorter cardio-pulmonary bypass times, shorter postoperative stays, decreased reintervention rates and lower health care costs [65]. Robust data are still needed to support these proposed outcomes.

## Figures and Tables

**Table 1:** Characteristics of different imaging modalities [5] (licensed under the Creative Commons Attribution 4.0 International License).

	2D Echo	3D Echo	Cardiac Catheterisation	CT	CMR
Radiation	-	-	++	+(+)	-
Temporal resolution	<5 ms	20–200 ms	1–10 ms	50–135 ms	20–50 ms
Spatial resolution	0.5–2.0 mm		0.3–1.2 mm	0.5 mm	0.8–2.0 mm
Quantitative ventricular function	++	++	+	++	+++
Ventricular volumetric	+	++	-	+	+++
Flow in vessels	+	-	+	-	+++
3D whole heart imaging	-	++	++	+++	+++
Atrioventricular valve assessment	++	+++	+	+	++
Semilunar valve assessment	++	+++	+	++	+++
Myocardial tissue characterisation	++	+	+	+	+++
Pressure measurements/estimation	+++	-	+++	-	++

The symbols -, +, ++, +++ indicate the degree to which the imaging modality provides information on each variable. The symbol - indicated no information and the symbols +,++, +++ indicate increasing amounts of information based on the original authors’ views.

## Data Availability

Not applicable.

## Supplementary Materials

The following supporting information can be downloaded at: https://www.mdpi.com/article/10.3390/jcdd9080269/s1, Video S1: Parachute Mitral valve with complex subvalvar stenosis, Video S2: DORV, Video S3: significant subaortic stenosis, Video S4: Superior Sinus Venosus ASDs before and after stenting.
